# Exploring the Digital Health Landscape: How adolescents living in urban and rural Vanuatu use social media to access health information

**DOI:** 10.12688/openreseurope.19670.1

**Published:** 2025-05-28

**Authors:** Krestina L. Amon, Guillaume Wattelez, Akila Nedjar-Guerre, Rowena Forsyth, Louisa R. Peralta, Marie-Jeanne Urvoy, Corinne Caillaud, Olivier Galy

**Affiliations:** 1 Cyberpsychology Research Group, The University of Sydney, Sydney, New South Wales, Australia; 2 Faculty of Medicine and Health, The University of Sydney, Sydney, New South Wales, Australia; 3 Interdisciplinary Laboratory for Research in Education, University of New Caledonia, Noumea, EA7483, New Caledonia; 4 Service Unit, University of New Caledonia, Noumea, New Caledonia; 5 Sydney School of Education and Social Work, The University of Sydney, Sydney, New South Wales, Australia; 6 Charles Perkins Centre, The University of Sydney, Sydney, New South Wales, Australia

**Keywords:** Melanesia, Pacific, adolescents, health information, digital health, social media

## Abstract

**Background:**

Investigating the use of online platforms by adolescents living in the Pacific Islands is important to understand how they navigate online resources to make informed decisions about their health. This study explores the use of online platforms, by adolescents in Vanuatu for health-related purposes.

**Methods:**

A total of 197 students (58% from an urban school and 42% from a rural school) completed a survey which collected quantitative and qualitative data about their use of digital technologies for health.

**Results:**

Results show that 77% of participants owned a mobile phone, which was mostly used to listen to music (34%) and play games (22%). Only 24% (n= 47) reported to have used apps, social media or websites for their health. Social media was the preferred category to use for health information, among both urban (25%) and rural (11%) participants, with Facebook, TikTok, YouTube, Instagram, and Twitter being the most frequently mentioned platforms. Reasons included, to gain knowledge, watch videos, chat with friends and look at posts. To search for health information, social media was more commonly used by rural students (12%) compared to urban students (8%). Conversely, search engines were more popular among urban students (12%) than rural students (5%). For discussing health topics online, social media was the predominant platform in both urban (9%) and rural (8%) areas.

**Conclusions:**

While reports often suggest a digital divide between urban and rural areas, results from our study challenge this with our findings showing similarities in the use of online platforms for health information between urban and rural adolescents in Vanuatu. Our paper considers the influencing factors of social media use for health-related purposes, reflects on cultural sensitivity, identifies the risks of misinformation and regards the role of policy and education as essential for effectively engaging this population with digital health tools, to promote positive health outcomes.

## Plain Language Summary

Young people are avid users of digital technology for information, but there is not a lot of research on their specific needs and how they use this technology for health in Vanuatu. With many sources available, it is important for researchers, healthcare providers, and educators to understand how adolescents in Vanuatu use online technology for health information. Our study found that in both urban and rural areas of Vanuatu, adolescents prefer using social media compared with other digital tools to access and discuss health information. These findings can help guide the inclusion of digital health literacy in school programs, improving knowledge in this group. Although only a small number of adolescents reported using digital health tools, their patterns of use are important as they highlight the need for tailored initiatives and help understand digital health trends among adolescents in Vanuatu.

## Introduction

In the contemporary digital landscape, adolescents' use of social media platforms has become a common avenue for accessing and sharing personal health-related information ( Hausmann *et al.*, 2017; Moreno *et al.*, 2018). Adolescents worldwide increasingly engage with online platforms as sources to search and discuss information related to health, marking a significant shift in their information-seeking behaviours ( Gray *et al.*, 2005; Skinner *et al.*, 2003). Adolescence marks a pivotal stage for establishing lifelong health habits ( Patton *et al.*, 2016), and as such, understanding their health information-seeking behaviours in various contexts is important. Studies show that adolescents engage and respond well to tailored digital education programs targeting health-related behaviour such as physical activity in Australia ( Caillaud *et al.*, 2022) and the Pacific Islands ( Galy *et al.*, 2019).

The Pacific Islands Countries and Territories (PICTs) consist of 22 islands in the world's largest region, the South Pacific ( Craig *et al.*, 2022). Over several decades, there has been a rapid socio-economic transition in the South Pacific region, characterised by modernisation, urbanisation, increased imports, and the digitisation of urban, peri-urban, rural, and tribal areas ( Bertrand-Protat *et al.*, 2024; Galy *et al.*, 2020; Wattelez *et al.*, 2024a). The process of digital transition within these PICTs has taken time to unfold ( Cave, 2012). Presently, there is a need to understand the relationship between adolescents and their use of media and digital environments. Literature addressing this topic is limited, with little evidence in the Pacific Islands. In countries like New Caledonia, a high number of digital devices are available in households with wide disparities across urban and rural areas related to the ongoing digital development of the country ( Nedjar-Guerre *et al.*, 2023). However, little is known about the use of digital technologies for health information by adolescents in Vanuatu, particularly comparing urban and rural areas.

With over 80 islands, Vanuatu is home to an estimated population of 328,928 people ( https://worldpopulationreview.com/countries/vanuatu). Linguistically diverse, English, French and Bislama are the official languages spoken but also have 138 indigenous Oceanic languages ( Crowley, 2000). The interaction between traditional practices and contemporary influences shapes the health experiences of the adolescent population in Vanuatu. Like many adolescents around the world, adolescents in Vanuatu engage with the Internet, social networking sites, and other mobile app technology, making digital technology a promising avenue for health. However, unlike adults, adolescents may still be in the process of accumulating the skills necessary to gather and evaluate reliable health information online ( Taba *et al.*, 2022). As such, the importance of their digital health literacy emerges as a pivotal factor for cultivating positive adolescent health and well-being outcomes. Digital health literacy represents the capacity to proficiently navigate health-related information and resources within digital environments ( Schulenkorf *et al.*, 2021) and warrants attention to address contemporary health challenges among this Pacific demographic. However, before this, it is crucial to gain insight into the current state of their online activities and behaviours related to health. Gaining insight into the patterns of use and purposes behind their engagement with online platforms for health-related information enables the development of effective educational initiatives and interventions that are tailored to their needs and preferences. This approach ensures that efforts to enhance digital health literacy are relevant and impactful for adolescents.

Access to, and the use of, digital technologies vary across urban, rural, and tribal areas. With higher population density and greater investment, urban areas generally have better access to healthcare services, digital infrastructure, and technology. Adolescents in these areas can benefit from a wider range of digital health tools, including mobile health apps and online health information resources. The challenge lies in ensuring the quality and reliability of the information accessed ( Swire-Thompson & Lazer, 2020), as well as addressing issues related to data privacy and security ( Razi *et al.*, 2020). Additionally, adolescents living in urban areas may face different health issues compared with their rural and tribal counterparts, necessitating tailored digital health (literacy) interventions ( Jacobs, 2020; Miller *et al.*, 2018).

For adolescents living in rural areas, digital health technologies can bridge the gap caused by geographical isolation ( Wang *et al.*, 2011). Telehealth services, for instance, can enable adolescents to access medical consultations without the need for long and often difficult travel to urban centers. However, the digital divide remains a significant barrier, with limited availability or access to resources and infrastructure for reliable internet connectivity in combination with limited digital literacy posing challenges to the effective use of technologies ( Ji *et al.*, 2024; Watson & Fox, 2021; Ye & Yang, 2020). Efforts to improve infrastructure and provide digital education are crucial to maximising the benefits of digital health solutions in these regions.

Furtherstill, residents in tribal areas present a unique context where traditional practices and beliefs play a significant role in healthcare. Integrating digital health technologies in these areas requires a culturally sensitive approach that respects and incorporates traditional health practices ( Galy *et al.*, 2022). Community engagement and collaboration with tribal leaders are essential to foster trust and acceptance of digital health solutions. Digital tools may be used to document and preserve traditional health knowledge, creating a valuable resource for both current and future generations.

This study therefore aims to explore the health information-seeking behaviours of adolescents in Vanuatu and identify their preferred online platforms for fulfilling these needs. Recognising the unique cultural and contextual factors in Vanuatu, this study aims to shed light on how adolescents living in urban areas compared with those living in rural areas navigate online resources to make informed health decisions. To address this aim, data from the current study will be analysed to answer which websites, social media platforms or apps, were i) their favourite to use for health, ii) used to search for health information, and iii) used to discuss health topics. By understanding their approach to seeking health information, we aim to bridge gaps in understanding and potentially tailor interventions that resonate with the specific needs of adolescents in Vanuatu, contributing to the advancement of their overall health outcomes.

## Methods

### Setting

This study was a part of the European *Family Farming, Lifestyle and Health in the Pacific* (FALAH) project (n° 873185) ( Fotsing & Galy, 2019). As such, Ethics approval for FALAH projects including fields of research in Fiji, New Caledonia, Solomon Islands, and Vanuatu was obtained from the Consultative Ethics Committee of New Caledonia (CCE 2018-06 001 and 2023-10 012). This study was conducted in accordance with the requirements of the Declaration of Helsinki ( World Medical Association [WMA], 2024).

Vanuatu archipelago is located in the South Pacific Ocean and consists of more than 80 islands. The two schools involved were selected based on the following criteria: (1) location, i.e. rural/urban; (2) island location, i.e. Esperitu Santo/Efate (amongst the most populated islands); (3) class grades, i.e. from levels/years 9 to 11; and (4) sufficient student numbers in the targeted class grades (n ≥ 150). The study was authorised by the Vanuatu Ministry of Education and the selected schools were contacted to present the research design to the staff in charge of the establishment. Before data collection, potential student participants and their parents were given written information about student involvement and details of the research study. This provided them an opportunity to opt out of participation in the study.

### Procedure and participant sample

***School recruitment*.** To understand the cultural context, members of the University of Sydney research team travelled to Vanuatu in October, 2022. During this trip, they visited two schools which were identified through existing connections with the FALAH network of international scientific teams ( https://falah.unc.nc/en). During the school visits, research team members met with the principals and deputy principals and explained the details of the research study and the delivery of the project workshops. The meeting included discussions about the school curriculum and student environment to understand the educational context and a tour of the school grounds. Following the initial meeting, the principals reached out to the research team about their interest in involving their students. From this visit, both schools signed up to participate in the study.

***Data collection*.** In June 2023, members from the University of Sydney and the University of New Caledonia research teams travelled to Vanuatu to run the project for data collection. The classes and students at each school were selected based on teacher interest and consent to participate in the study.

A participant information template was sent home with the students, providing parents with information about the study, and an opportunity to opt-out of the study if they did not want their child to participate. If no signed note was returned, it was understood as consenting to involvement in the study. For parents who were living in remote, isolated islands and were unable to sign and return the forms due to illiteracy, we obtained verbal consent from community authorities (directors, teachers, and Kustom leaders). In research, consent is sought from both parents and adolescents for studies involving vulnerable populations, including young people. However, in Vanuatu when parents are not available, such as in the case of students residing in boarding schools or living in remote islands, in line with local customs it is common practice that a representative of authority (for example a teacher, school director, or Kustom leader) can approve the ethical aspects of the study. The principals of the participating schools provided consent to involve their students in our study. In instances where written consent was not feasible, oral or verbal consent was accepted. The students whose parents did not opt them out of the study, had consent from community authorities, but did not want to participate, verbally expressed this to their teacher or a research team member and were free to leave the classroom without participating in the study. All adolescents provided individual written consent through the submission of the online survey.

Students completed a questionnaire either offline via a REDCap mobile app on digital tablets or online via the same REDCap questionnaire on a computer in a classroom. The questionnaire collected both quantitative and qualitative data. Depending on the student’s preferred language, the questionnaire could be completed in either French or English. It is recognised that these languages may not be the student’s first language, which is likely to be Bislama, however, it was a requirement that they could read and write in either French or English, as taught in their school curriculum. During the completion of the questionnaire, teaching staff and researchers remained in the classroom in the event students required support to better understand any of the questions.

### Data analysis

This study is a part of a larger research project aiming to better understand the lifestyle and behaviors of adolescents living in the PICTs and mainly focused on physical activity, diet and the use of digital technologies ( Galy *et al.*, 2024a; Galy *et al.*, 2024b). Selected questions from the digital technology component of the questionnaire about the use of digital technology were adapted from an Australian survey ( Armstrong *et al.*, 2021) and from a previous study conducted in New Caledonia that explored the impact of digital screen time on unhealthy food consumption ( Nedjar-Guerre *et al.*, 2023). Results from the New Technologies and Medias and Digital Tools and Health sections of the questionnaire were analysed and this study presents the descriptive statistics and qualitative findings of digital technology use for health by adolescents living in Vanuatu.

***Quantitative data*.** The use of digital tools was assessed through the following closed questions: 1) When you are on the computer and/or tablet, what type of actions do you most often performs? 2) When you are on the mobile phone, what type of actions do you most often perform? and 3) Do you use apps, social media or websites for your health? Responses to these questions, as well as the open questions that were categorised, are presented with percentages and shown with barplots.

***Qualitative data*.** Within the survey, the following open questions were used to better understand participants’ use of digital tools regarding health information: (1) “Which social media platform/site is your favourite to use for health? And what do you like about it?”, (2) “For searching health information, which app, social media or website do you use?” and (3) “For discussing health topics, which app, social media or website do you use?”

Where required, responses were first translated from French to English and then a simple content analysis was performed. Responses were assigned an open code that named the platform in that response. Similar codes were grouped under axial codes and given a descriptive heading. For example, “Facebook” was grouped with “TikTok” under “Social Media”, and “Google” was grouped with “Chrome” under “Search engine”. KA and GW reviewed all coding and discussed the final codes. There were no disagreements requiring further discussion.

## Results

### Participants

A total of 245 participants were recruited from two schools in Vanuatu. Of these, 197 participants completed the questionnaire. Majority of participants from both locations were female (62% in the urban school and 66% in the rural school; Table 1).

**Table 1.** Number of participants according to school location and sex.

	Number of participants	Average age (sd)
**Urban**	114	16.3 (1.27)
Female	63	16.1 (1.23)
Male	51	16.4 (1.32)
**Rural**	83	15.4 (0.82)
Female	53	15.4 (0.78)
Male	30	15.5 (0.91)

### Device use

A total of 79.8% (n= 91) of participants from the urban location and 72.3% (n= 60) from the rural area reported to have owned a mobile phone. From this, 25.3% (n= 23) of participants from the urban location and 6.6% (n= 4) from the rural location shared the phone with other people.

**Figure 1.**
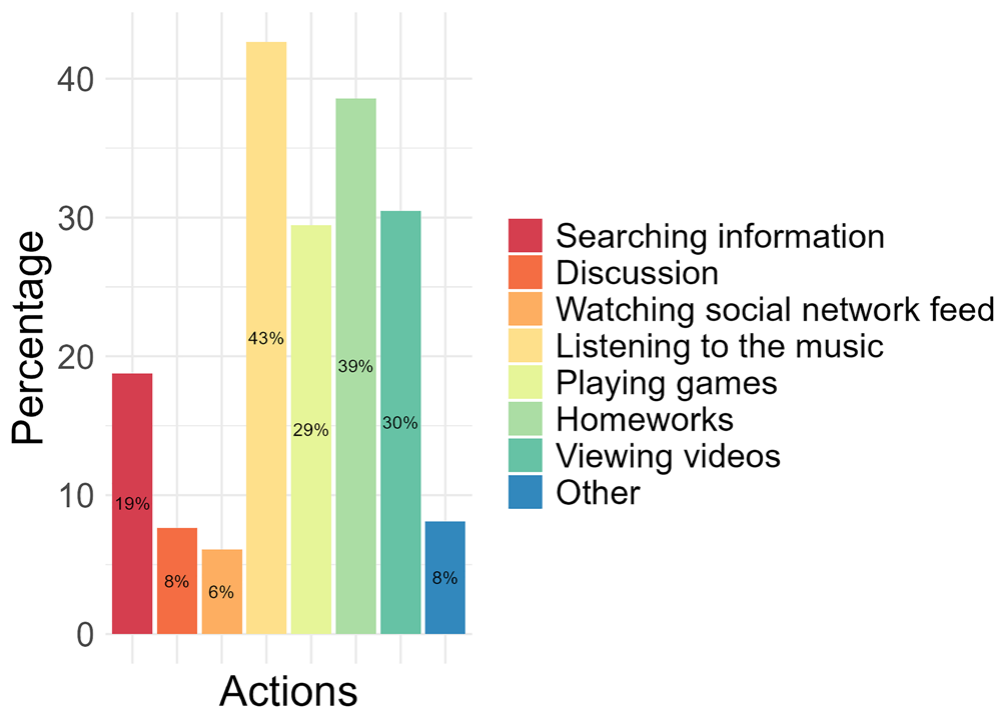
The most performed activities conducted on the computer and/or tablet by participants.

Of the activities conducted on a computer or tablet, 43% of the participants identified “listening to the music” as the most performed, followed by completing “homework” (39%), “viewing videos” (30%), and “playing games” (29%). The least performed activities conducted by participants on a computer or tablet were “watching social network feed” (6%), “discussion” (8%), and “other”(8%), which were not specified ( Figure 1).

**Figure 2.**
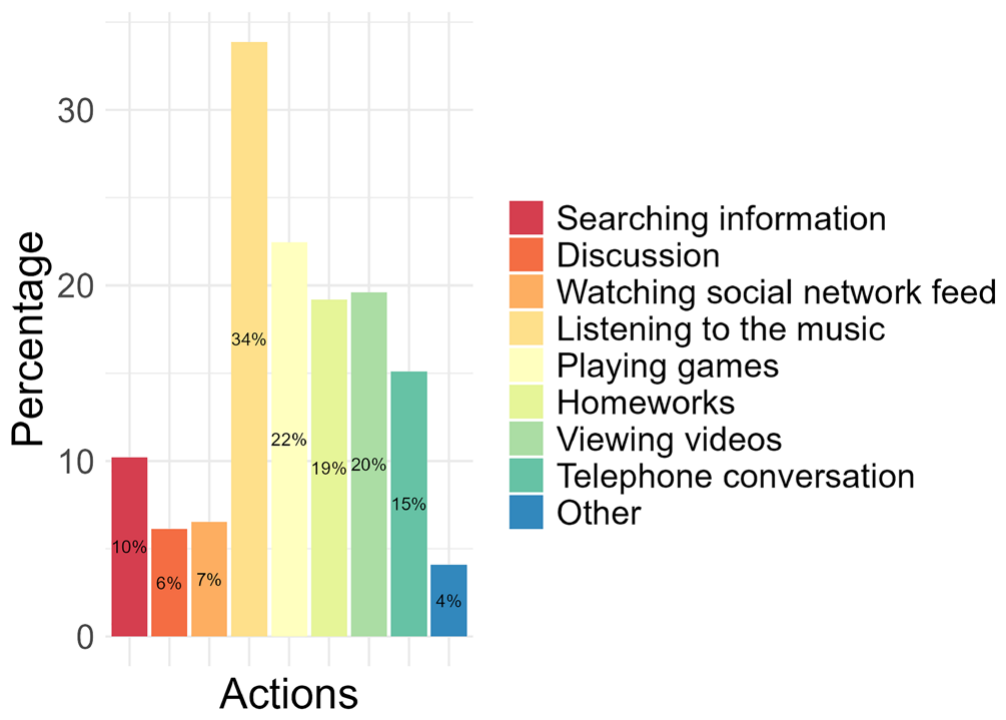
The most performed activities conducted on the mobile phone by participants.

Of the activities conducted on a mobile phone, 34% of the participants identified “listening to the music” as the most performed, followed by completing “playing games” (22%), “viewing videos” (20%), and “homeworks” (19%). The least performed activities conducted by participants on a mobile phone were “watching social network feed” (7%), “discussion” (6%) and “other”(4%), which were not specified ( Figure 2).

**Figure 3.**
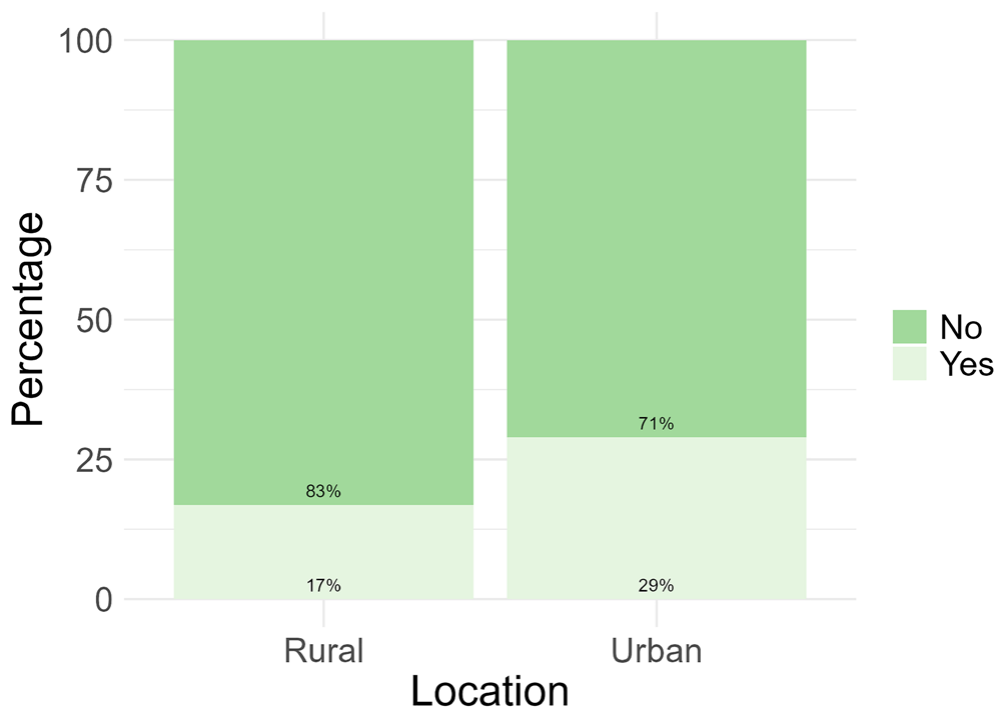
Participant use of apps, social media, and websites for health information by location.

Only 24% (n = 47) of the total number of participants reported to have used apps, social media or websites for their health. However, when analysed based on location, more participants from the urban location reported to have used apps, social media or websites for their health (29%, n= 33) ( Figure 3).

### Qualitative analysis

Of the 197 number of total participants who completed the questionnaire, 24% of participants reported that they used apps, social media, or websites specifically for their health. To find out which platforms were: i) their favourite to use for health, ii) used to search for health information, and iii) used to discuss health topics, participants were asked open-ended questions and a simple content analysis was performed. The following results present the qualitative findings between the urban and rural areas.

**Figure 4.**
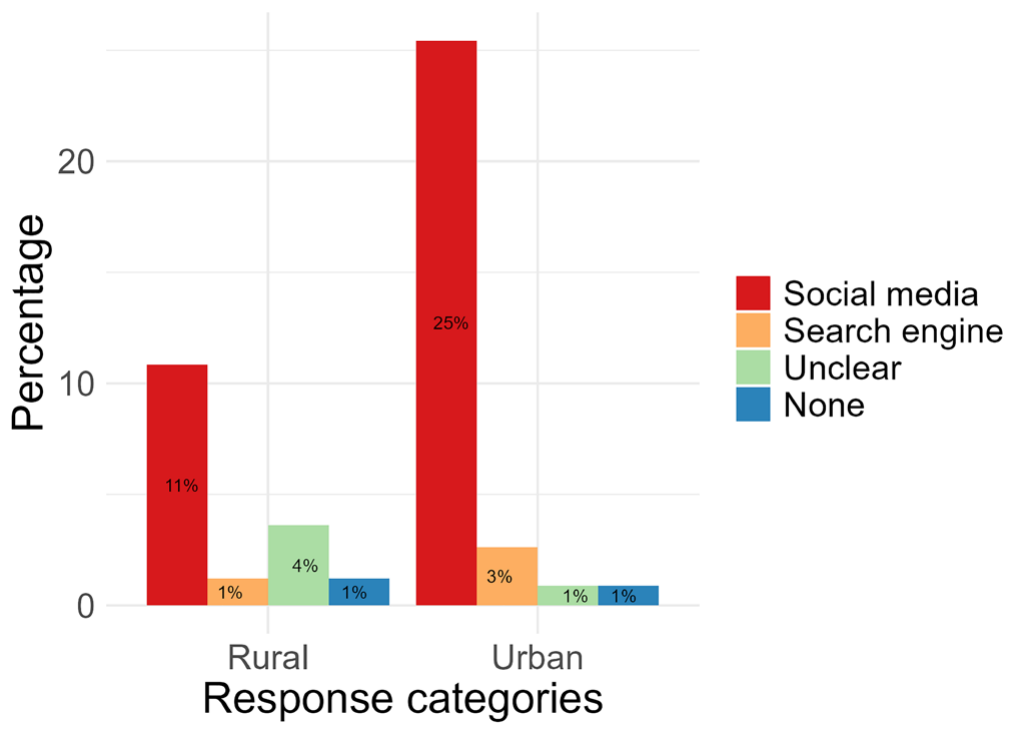
Comparing rural and urban participant preferences for using online platforms for health.

When asked the question “Which social media platform/site is your **favourite to use for health? And what do you like about it?**”, “social media” was found to be participants' favourite for both urban (25%, n= 29) and rural locations (11%, n= 9). Platforms within this code included Facebook (n= 20), TikTok (n= 16), YouTube (n= 5), Instagram (n= 2), and Twitter (n= 1). A total of 3% (n= 3) of urban location responses and 1% (n= 1) of rural location responses were categorised under “Search engine” and these included “Google” (n= 3) and “Chrome” (n= 1) responses. Responses categorised under “Unclear” (1%, n= 1, from urban and 4%, n= 3 from rural) included responses which could not be translated into English or did not explicitly name a platform or website to answer the question ( Figure 4). From these responses, only 7 included reasons what they liked about these platforms. Reasons were summarised and categorised into “Gain knowledge” (71%, n= 5), “Watch videos” (43%, n= 3), and “Other” which included to “chat with friends” and “look at posts” (29%, n= 2). Two responses (1 from urban and 1 from rural) either did not type a response despite stating they used social media platforms or websites for health.

**Figure 5.**
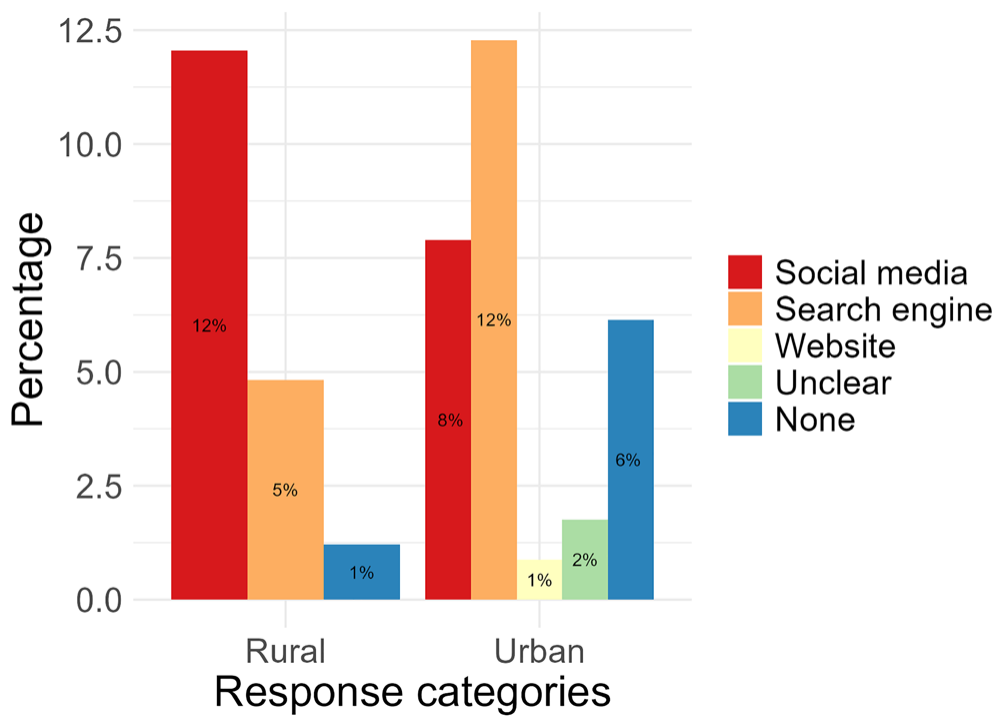
The preferred use of types of online platforms by rural and urban participants’ to search for health information.

Following analysis of the question “For **searching health information**, which app, social media or website do you use? “social media” was found to be the most used in the rural area (12%, n= 10) compared with the urban area (8%, n= 9). Platforms within the social media code included Facebook (n= 9), TikTok (n= 3), and YouTube (n= 7). The most used in the urban area was found to be “search engines” (12%, n= 14) compared with the rural location (5%, n= 4). Responses for these included “Google” (n= 16), “Chrome” (n= 1) and “I’m usually looking for, regardless of the website” [translated from: “Je cherche en générale , peut importe le site”] (n= 1). Only one participant from the urban location (1%) responded with “websites”. There were 2 responses from the urban location that could not be translated into English or did not explicitly name a platform or website, and were categorised under “Unclear” (2%). Participants from both the urban (6%, n= 7) and rural (1%, n= 1) locations indicated responses but either did not type a response or responded with “I don’t use” despite stating they used social media platforms or websites for health, but may not use it specifically to search health information ( Figure 5).

**Figure 6.**
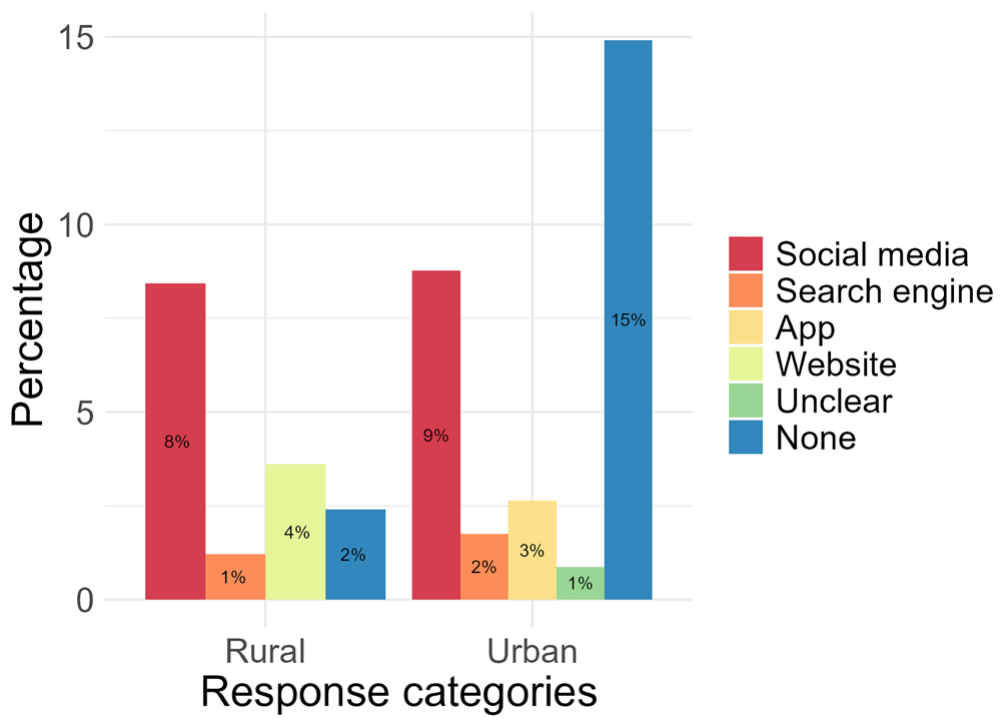
Types of online platforms preferred by rural and urban participants to discuss health topics.

When asked the question “For **discussing health topics** with other people online, which app, social media or website do you use?” “social media” was found to be the most used in both urban (9%, n= 10) and rural (8%, n= 7) locations. Platforms within this code included Facebook (n= 13), Instagram (n= 1), Twitter (n= 1), YouTube (n= 2), and Snapchat (n= 1). Two responses from the urban location (2%) and one response (1%) from the rural location were categorised under “Search engine” and these included “Google” (n= 3) responses. Only three participants in the urban location (3%) identified “Apps” and these included chat apps including “Messenger” (n= 2) and a “Food app” (n= 1) responses. “Website” was also identified by four participants from the rural location (4%). There was only one response (1%) which was categorised under “Unclear” and included a response that could not be translated into English from the urban location. Participants from both the urban (15%, n= 17) and rural (2%, n= 2) locations provided responses which either did not type a response or responded with “I don’t use” despite stating they used social media platforms or websites for health, this may suggest they do not use it to discuss health topics ( Figure 6).

## Discussion

Our study collected 197 completed questionnaires from adolescents in Vanuatu regarding their engagement with digital technology for health-related purposes. However, only 24% of participants reported using apps, social media, or websites for their health. Despite comprising a minority within the surveyed population, the significance of this subset cannot be understated. While reports often suggest a digital divide between urban and rural areas, the results from our study among adolescents in Vanuatu challenge this notion. Our current findings demonstrate that of the participants who reported having used digital technology for their health indicated similar responses across both urban and rural areas, suggesting that with access, there may not be a digital divide.

The analysis of findings from these adolescents reporting the use of digital technologies for their health holds importance as it provides insights into the health information-seeking behaviours of adolescents in Vanuatu. Understanding the preferences identified by this group serves as a pivotal point for targeted strategies to assist them with making appropriate health decisions. Tailoring educational initiatives, and resources to align with the needs and preferences of this population is required for effective engagement of digital and online health tools and positive health outcomes.

### Influencing factors of social media use for health-related purposes by adolescents in Vanuatu

The type of device used plays a crucial role in shaping how adolescents in Vanuatu engage with health-related digital content. Smartphones are likely the most commonly used devices due to their portability and multifunctionality, allowing adolescents to easily access social media apps, watch videos, and interact with online communities. In this study, the participants used their mobile phones mostly for music-related activities and less likely to search for information or use social media. Computers and tablets, while less portable, offer a larger screen that enhances the length and viewing experience of content consumption. Completing homework was the second most reported activity to be conducted on these devices in the current study, following music-related activities. The device type can also affect the format of content that adolescents prefer. Short videos and quick updates particularly on social media platforms are more suited to smartphones, while websites and documents might be better viewed on tablets or computers.

Previous studies have shown that online search engines was the most common tool to use to actively search for health information ( Freeman *et al.*, 2018). In this current study, the use of search engines was more popular to search for health information in the urban location compared with the rural location, where rural participants preferred to search health information through social media platforms. Social media was also identified as the favourite to use for health-related activites and discussing health topics as identified by adolescents from both urban and rural areas. This aligns with current literature that adolescents from other regions use social media platforms to access health-related information, including topics such as mental health, sexual health, substance use, and specific medical conditions. Popular platforms including Facebook, Twitter (now known as X), and YouTube act as accessible reservoirs of diverse knowledge, empowering adolescents with important health information ( Moreno *et al.*, 2018; Yonker *et al.*, 2015). Social media has also been found to serve as a space for peer interaction and influence regarding health behaviours and attitudes ( Yonker *et al.*, 2015). Fostering a sense of community engagement, social media enables adolescents to share personal health experiences, practices and solutions within a digitally interconnected space. These findings have leveraged the potential social media has as a platform to delivering health interventions, with positive findings ( Alvarez-Jimenez *et al.*, 2020; Amon *et al.*, 2022).

These findings were revealed by only a quarter of the total participants, however, this may be influenced by multifaceted factors that are characteristic of the Pacific Islands. Whilst there were no specific differences in use between rural and urban locations, the limited accessibility of internet infrastructure, coupled with socioeconomic disparities may significantly impact the opportunity to use digital technologies for these purposes. In addition, the amalgamation of traditional cultural norms with the current digital landscape shapes the acceptance and credibility of health information that is disseminated through these online platforms. The interactive nature of these platforms encourages open dialogues, enhancing health literacy and promoting discussions around taboo health topics in a culturally sensitive manner ( Chen, 2023). Understanding these multifaceted and complex connections is crucial for a comprehensive understanding of adolescent use of social media for health-related purposes within the Vanuatu context.

### Cultural sensitivity

Culture may affect health by shaping and influencing habits and behaviours related to precursors of health, development of disease, and by controlling their physical environment ( Fleisher *et al.*, 2013). Interpretations of health and illness may vary with different cultural groups and ultimately inform why some cultural groups may choose to adopt or not adopt preventative health behaviours. Thus, health education materials need to be culturally appropriate. An important component of culture is language, and the impact of language on minority subgroups differs based on social and demographic characteristics. A lack of understanding of written English or French is a barrier that can cause misunderstandings that lead to inappropriate health decisions ( Sauni & Neal, 2012).

The rapid dissemination of potentially culturally insensitive information is of concern. Vanuatu is culturally diverse which amplifies the susceptibility to misinterpretation of health-related content available online, and the lack of digital health resources in local languages can limit the reach and effectiveness of health initiatives.

### Risks

***Misinformation*.** Whilst social media serves as a means to access health information by adolescents in Vanuatu, it is unregulated which presents potential risks of misinformation ( Suarez-Lledo & Alvarez-Galvez, 2021; Swire-Thompson & Lazer, 2020). Taba *et al.* (2022) explored the relationship between adolescents’ self-efficacy and digital health literacy, emphasising the critical role these factors play in navigating online health information. Thus, while adolescents increasingly turn to social media for health-related information, their ability to discern credible sources from misinformation is often limited by their digital health literacy skills, emphasising the risk of misinformation, particularly in contexts like Vanuatu, where digital literacy education may be less prevalent. Adolescents with higher self-efficacy and better digital health literacy are more adept at evaluating the trustworthiness and relevance of online health content, thereby reducing their susceptibility to misinformation. Therefore, there is a need for targeted educational interventions and policies to enhance digital health literacy among adolescents, ensuring they can safely and effectively use social media for health information.

***Exposure*.** There is evidence of passive health information exchange via social media ( Raeside *et al.*, 2022). Findings from this current study show that adolescents in Vanuatu engage in several other activities on their devices. Thus, even if an adolescent is not actively searching for health-related information, their presence on online websites and social media leaves them open to a variety of unsolicited unhealthy promotional content. This exposure can significantly influence adolescents’ health behaviours and perceptions. Potvin Kent *et al.* (2024) found that youth exposed to digital marketing of unhealthy foods, including fast food and sugary drinks, were more likely to consume these foods and more frequently. The constant exposure to promotional content can lead to increased consumption of unhealthy foods, contributing to poor dietary habits and related health issues. This highlights the importance of digital health literacy in helping adolescents critically evaluate the online health information they encounter, whether intentionally sought or not. Without adequate digital literacy skills, adolescents are more susceptible to the persuasive tactics of digital marketing, which can exacerbate unhealthy behaviours and the risk of misinformation.

### The role of policy and education

Policy and education play a crucial role in shaping adolescents’ use of social media for health-related purposes. There is a required focus on equipping adolescents with the digital literacy skills needed to critically evaluate health information encountered online, consequently reducing the risk of misinformation ( Taba *et al.*, 2022; Wendt *et al.*, 2023). Educational interventions, both within school settings and through community-based programs, play a significant part. For example, integrating digital literacy into the school curriculum can help students develop the necessary competencies to navigate the digital landscape effectively, whilst also providing them with free connectivity access. Also providing school staff such as onsite school nurses with such skills as additional resources. Additionally, community workshops and campaigns led by non-government organisations can provide practical guidance on identifying credible sources and understanding the potential impacts of social media on health behaviours. By fostering a supportive environment that encourages informed and responsible social media use, these policy and educational efforts can significantly influence adolescents’ health outcomes. Further still, ongoing evaluation and adaptation of these programs are essential to address the evolving digital habits and health needs of adolescents in Vanuatu.

### Limitations and future research directions

This study serves as an important direction to determine the online activities conducted by adolescents to access health information and communicate about health topics, between urban and rural areas in Vanuatu. However, a limitation is the low number of participants who completed the open-ended questions, which may affect the generalisability of the findings. Having a greater number of participant responses would provide a more comprehensive understanding by identifying more online platforms, apps, and specific websites and yield more detailed insights into the patterns and preferences of adolescents in this context, further identifying any differences between the urban and rural settings.

## Conclusion

Adolescents are prominent users of information promoted through digital technology, however, there exists a noticeable gap in research concerning their specific information needs and patterns of use, particularly within the Vanuatu context. With a multitude of information sources available to them, it is important for researchers, healthcare providers, and educators to gain insight into how adolescents in Vanuatu use online technology to fulfill their health information needs. Our current findings show that of the digital tools used by adolescents, there was a preference for the use of social media by adolescents residing in both urban and rural locations in Vanuatu to access health information and discuss health issues. These findings may be instrumental in informing decisions regarding the integration of digital health literacy resources into educational curricula, thereby contributing to the advancement of knowledge within this population. Thus, while these findings may represent a numerical minority, their usage patterns and behaviours in engaging with digital health platforms underscore their significance in shaping tailored initiatives and furthering our understanding of digital health trends among adolescents in Vanuatu.

## Ethics and consent

This study was a part of the European Family Farming, Lifestyle and Health in the Pacific (FALAH) project (n° 873185) ( Fotsing & Galy, 2019). As such, Ethics approval for FALAH projects including fields of research in Fiji, New Caledonia, Solomon Islands, and Vanuatu was obtained from the Consultative Ethics Committee of New Caledonia (CCE 2018-06 001 and 2023-10 012). This study was conducted in accordance with the requirements of the Declaration of Helsinki ( World Medical Association [WMA], 2024).

A participant information template was sent home with the students, providing parents information about the study, and an opportunity to opt-out of the study if they did not want their child to participate. If no signed note was returned, it was understood as consenting to involvement in the study. For parents who were living in remote, isolated islands and were unable to sign and return the forms due to illiteracy, we obtained verbal consent from community authorities (directors, teachers, and Kustom leaders). In research, consent is sought from both parents and adolescents for studies involving vulnerable populations, including young people. However, in Vanuatu when parents are not available, such as in the case of students residing in boarding schools or living in remote islands, in line with local customs it is common practice that a representative of authority (for example a teacher, school director, or Kustom leader) can approve the ethical aspects of the study. The principals of the participating schools provided consent to involve their students in our study. In instances where written consent was not feasible, oral or verbal consent was accepted. The students whose parents did not opt them out of the study, had consent from community authorities, but did not want to participate, verbally expressed this to their teacher or a research team member and were free to leave the classroom without participating in the study. All adolescents provided individual written consent through the submission of the online survey.

## Data Availability

The data that support the findings of this study are openly available in Zenodo (open – Creative Commons Attribution 4.0 International – CC-BY 4.0). Exploring the Digital Health Landscape: How adolescents living in urban ans rural Vanuatu use social media to access health information (anonymized version). DOI: 10.5281/zenodo.15074015 ( https://zenodo.org/records/15074016) ( Wattelez *et al.*, 2025) Data are available under the terms of the Creative Commons Attribution 4.0 International Restricted version of the data that support the findings of this study are available in Zenodo (restricted). Exploring the Digital Health Landscape: How adolescents living in urban and rural Vanuatu use social media to access health information (non-anonymized version). DOI: 10.5281/zenodo.14323389 ( https://zenodo.org/records/14323390) ( Wattelez *et al.*, 2024b) For ethical reasons, the restricted data is not freely open because the data has not been anonymized. The risk of reidentification is not negligible. However, within reason, authenticated Zenodo users can request access to the data by contacting the authors through the Zenodo website, or by sending a direct email to dirlire@unc.nc. The authors will consider all serious requests. A basic condition to access to this dataset will be to certify that the requester complies with the European General Data Protection Regulation (GDPR) and the data will not be used to identify individuals. Data are available under the terms of the Creative Commons Attribution 4.0 International The full questionnaire is available on Zenodo. English: https://doi.org/10.5281/zenodo.14031903 ( Galy *et al.*, 2024a) French: https://doi.org/10.5281/zenodo.14183635 ( Galy *et al.*, 2024b) Data are available under the terms of the Creative Commons Attribution 4.0 International
