# Supplementary material for: Exploring the Digital Health Landscape: How adolescents living in urban and rural Vanuatu use online platforms to access health information
Source: Open Res Eur. 2025 Oct 24;5:148. Originally published 2025 May 28. [Version 3] doi: 10.12688/openreseurope.19670.3 (PMC12559845; doi:10.12688/openreseurope.19670.3)
Supplement: Peer review history

# Reviewer report: Reviewer response for version 3

**DOI:** 10.21956/openreseurope.23428.r62762

**Published:** 2025-11-21

**Authors:** Nicola Bruno

**Affiliations:** 1 University of Modena and Reggio Emilia, Province of Reggio Emilia, Italy

I would advise being more cautionary; for example, in the Abstract > Background, I'd say "preliminary study."

 In Abstract > Results, I'd point out that the number of participants is low; to confirm the findings, research with more participants should be conducted. 

 In Abstract > Conclusions, I'd say "our preliminary findings."

 I advise inserting these changes also in the text of the article

If applicable, is the statistical analysis and its interpretation appropriate?

Yes

Is the study design appropriate and is the work technically sound?

Partly

Is the work clearly and accurately presented and does it engage with the current literature?

Yes

Are the conclusions drawn adequately supported by the results?

Partly

Are sufficient details of methods and analysis provided to allow replication by others?

Partly

Are all the source data and materials underlying the results available?

Yes

Reviewer Expertise:

Media education, Information Literacy, Disinformation, Source evaluation, Fact-checking

I confirm that I have read this submission and believe that I have an appropriate level of expertise to confirm that it is of an acceptable scientific standard.

# Reviewer report: Reviewer response for version 3

**DOI:** 10.21956/openreseurope.23428.r62760

**Published:** 2025-11-17

**Authors:** Rasika Manori Jayasinghe

**Affiliations:** 1 Prosthetic Dentistry, Penang International Dental College (PIDC), Butterworth, Penang, Malaysia; 2 Prosthetic Dentistry, University of Peradeniya, Peradeniya, Central Province, Sri Lanka

i do not have further comments. thanks

If applicable, is the statistical analysis and its interpretation appropriate?

No

Is the study design appropriate and is the work technically sound?

Yes

Is the work clearly and accurately presented and does it engage with the current literature?

No

Are the conclusions drawn adequately supported by the results?

Partly

Are sufficient details of methods and analysis provided to allow replication by others?

Yes

Are all the source data and materials underlying the results available?

Yes

Reviewer Expertise:

OHRQOL, dental public health, prosthetic dentistry, implant dentistry

I confirm that I have read this submission and believe that I have an appropriate level of expertise to confirm that it is of an acceptable scientific standard.

# Reviewer report: Reviewer response for version 3

**DOI:** 10.21956/openreseurope.23428.r62763

**Published:** 2025-10-27

**Authors:** Kay Oddone

**Affiliations:** 1 Charles Sturt University, Albury, New South Wales, Australia

I approve these changes.

If applicable, is the statistical analysis and its interpretation appropriate?

Yes

Is the study design appropriate and is the work technically sound?

Yes

Is the work clearly and accurately presented and does it engage with the current literature?

Partly

Are the conclusions drawn adequately supported by the results?

Partly

Are sufficient details of methods and analysis provided to allow replication by others?

Partly

Are all the source data and materials underlying the results available?

No

Reviewer Expertise:

Education, digital literacy, information literacy.

I confirm that I have read this submission and believe that I have an appropriate level of expertise to confirm that it is of an acceptable scientific standard.

# Reviewer report: Reviewer response for version 2

**DOI:** 10.21956/openreseurope.23157.r61846

**Published:** 2025-10-17

**Authors:** Nicola Bruno

**Affiliations:** 1 University of Modena and Reggio Emilia, Province of Reggio Emilia, Italy

This study fills a significant research gap by examining the digital health information-seeking behaviors of adolescents in Vanuatu. The study's descriptive nature offers useful initial insights, but considerable methodological limitations hinder the generalizability and profundity of the findings.

**Strengths**

 New contribution: Addresses a significant research deficiency in comprehending digital health engagement within Pacific Island contexts.

 Comparison of two settings: Efforts to juxtapose urban and rural settings

**Main Issues**

**1. Size and Representativeness of the Sample**

 As underlined by the authors, the overall sample (n=197) is limited and may not accurately reflect the larger adolescent population in Vanuatu.

 The subgroup utilizing digital devices for health information is significantly small (24%, n=47), which severely restricts the generalizability of findings regarding digital health behaviors.

 Recommendation: Either significantly enlarge the sample or rearticulate the study objectives to more explicitly recognize these limitations in the title  and abstract.

**2. Questionnaire Design: Major Problems**

 2.1 Typology of questions: The questionnaire goes straight to digital platform use without first asking about people's general health information. A preliminary question like "Where do you usually get health-related information?" with options like non-digital sources (TV, radio, family, friends, teachers, healthcare providers, influencers) would help explain why so few people are using digital tools.

 In addition to usage, a question regarding trust would have been equally important: which sources are considered most trustworthy for health information?

 2.2 Structure of the questions: Questions 2 and 3 ("For searching health information..." and "For discussing health topics...") are too similar, so people are likely to give the same answers to both. This design flaw makes it harder to understand the different ways people can use digital health.

 2.3 Unexamined exposure phenomenon: The small number of teens who say they use digital media for health reasons (24%) probably doesn't include people who just passively see health content. The questionnaire must differentiate between:

- Actively looking for health information
- Passive exposure while doing other things (like listening to music or watching influencers’ videos, which is what most people do with their devices)

Suggestion: Add questions like, "Do you come across health-related content online even when you're not looking for it?" If so, where?; “Do you ever come across influencers or content creators discussing health-related topics? If so, where have you encountered them? Do you trust them?”; “Which sources do you consider most trustworthy for health information?”

**3. Claims in the discussion that don't have any evidence to back them up**

 Some parts of the discussion make claims about the Vanuatu context without backing them up with data from this study:

 - The "Policy and Education" section suggests adding digital literacy to school lessons, but the study didn't look at how well students can evaluate online information, how confident they are in doing so, or what health resources are already available in schools.

 - The "Misinformation" section goes into great detail about the dangers of unregulated social media and how teens aren't very good at telling which sources are reliable, but there were no questions that asked students about their own experiences with conflicting or unreliable health information.

 Suggestion: Either include a short follow-up study to confirm these discussion points or clearly label these sections as "implications from the literature" instead of "interpretations of the current findings." Or, say these directly as hypotheses that need to be tested in the future.

**4. Introduction**

 The justification for the specific selection of Vanuatu should be better developed. The authors acknowledge the scarcity of research, which is applicable to numerous Pacific Island nations. Advice: Give specific reasons why Vanuatu is especially important in this context (for example, its linguistic diversity, its unique digital infrastructure problems, its unique cultural health practices, etc.).

**5. Discussion**

 Several discussion sections offer literature reviews that lack a definitive link to the study findings:

 "Policy and Education" part: Discusses the role of schools and digital literacy programs but presents no empirical data from this study about

 Sections on "Misinformation" and "Exposure": These discussions are mostly theoretical and don't have any survey data to back them up in the Vanuatu context, even though they are important in theory.

**6. References**

 The Introduction asserts that "adolescents worldwide increasingly engage with online platforms," referencing Gray et al. (2005) and Skinner et al. (2003). Because social media and digital platforms have changed so much in the last 20 years, these citations should be backed up with more recent evidence (after 2018).

**More Ideas**

 - Comparative framing: Since there isn't much research on the Pacific Islands, you might want to think of this as the first in a series of cross-cultural comparisons. Clearly articulate the necessity for analogous studies in other Pacific nations to discern region-wide patterns as opposed to country-specific phenomena.

 - Qualitative depth: Since few participants gave reasons for their platform choices, think about whether follow-up interviews or focus groups could help you understand the "why" behind those choices better.

**Final remark**

 This study makes a valuable contribution by providing visibility to an underrepresented region and addressing a genuine research gap. Data from Vanuatu adolescents' digital health behaviors is important and deserves publication. However, a substantial revision to the manuscript's framing and discussion of findings is necessary.

 The study should be explicitly positioned as exploratory and hypothesis-generating—the first step in understanding this population—rather than as providing definitive evidence for policy recommendations. Given the small sample and particularly the very small subgroup engaging with digital health tools (24%, n=47), conclusions about digital literacy education, misinformation risks, and policy interventions are premature.

**Recommended approach:**

 - Reframe the study explicitly as preliminary/exploratory work that establishes baseline patterns and identifies questions for future research

 - Present this as the first in a series of investigations that will require larger, more representative samples; Follow-up qualitative studies to understand the "why" behind the patterns

 - Avoid prescriptive recommendations (e.g., integrating digital literacy into curricula) without empirical support from this study

 The data collected is valuable precisely because it highlights how little we know about this population. The authors should embrace this as groundwork rather than overextending their conclusions.

If applicable, is the statistical analysis and its interpretation appropriate?

Yes

Is the study design appropriate and is the work technically sound?

Partly

Is the work clearly and accurately presented and does it engage with the current literature?

Yes

Are the conclusions drawn adequately supported by the results?

Partly

Are sufficient details of methods and analysis provided to allow replication by others?

Partly

Are all the source data and materials underlying the results available?

Yes

Reviewer Expertise:

Media education, Information Literacy, Disinformation, Source evaluation, Fact-checking

I confirm that I have read this submission and believe that I have an appropriate level of expertise to confirm that it is of an acceptable scientific standard, however I have significant reservations, as outlined above.

# Reviewer report: Reviewer response for version 2

**DOI:** 10.21956/openreseurope.23157.r61116

**Published:** 2025-10-08

**Authors:** Rasika Manori Jayasinghe

**Affiliations:** 1 Prosthetic Dentistry, Penang International Dental College (PIDC), Butterworth, Penang, Malaysia; 2 Prosthetic Dentistry, University of Peradeniya, Peradeniya, Central Province, Sri Lanka

I accept the revised version including description of limitations in the discussion. However, conclusions need revision again. As an example, the first sentence in the conclusions is adolescents are prominent users of infor....however, to get it as a conclusion, there should be another study to compare them with other age groups. therefore, it cannot be used in conclusion. further, ' it is important for researchers, health care...' sentence is a recommendation or future use, not a conclusion too. therefore, improvement of conclusion with use of ' only the ' highlights in the study would be appropriate.

If applicable, is the statistical analysis and its interpretation appropriate?

No

Is the study design appropriate and is the work technically sound?

Yes

Is the work clearly and accurately presented and does it engage with the current literature?

No

Are the conclusions drawn adequately supported by the results?

Partly

Are sufficient details of methods and analysis provided to allow replication by others?

Yes

Are all the source data and materials underlying the results available?

Yes

Reviewer Expertise:

OHRQOL, dental public health, prosthetic dentistry, implant dentistry

I confirm that I have read this submission and believe that I have an appropriate level of expertise to confirm that it is of an acceptable scientific standard, however I have significant reservations, as outlined above.

# Response

**Published:** 2025-10-15

**Authors:** Krestina Amon

**Affiliations:** Faculty of Medicine and Health, The University of Sydney, Sydney, New South Wales, Australia

We thank the reviewer for considering our changes and the additional feedback regarding our Conclusion section. We have now reframed the wording of our Conclusion to focus more on our results, which then opens to future research. 

 The intention of our sentence, “Adolescents are prominent users of information promoted through digital technology” was to reflect the growing visibility of adolescents in digital spaces, particularly in relation to health information, as observed in our study. To avoid overgeneralisation, we have revised the sentence to more accurately reflect our findings (and those of the research cited in the Introduction to support it).

 Regarding the feedback of our sentence, “With a multitude of information sources available to them, it is important for researchers, healthcare providers, and educators to gain insight into how adolescents in Vanuatu use online technology to fulfill their health information needs”, this sentence was intended to contextualise the relevance of our study and highlight the rationale behind our research focus, rather than to serve as a recommendation for future action.

 However, we understand that you have read it as a forward-looking statement. To address this, we have revised the sentence to better reflect its role in summarising the significance of our findings. However, we believe it is justified to keep our future exploration recommendations as our final sentence in the Conclusion in addition to our key findings, as this was discussed and common in the Conclusion section of research papers published.

# Reviewer report: Reviewer response for version 1

**DOI:** 10.21956/openreseurope.21277.r58191

**Published:** 2025-09-05

**Authors:** Philomina Pomaah Ofori

**Affiliations:** 1 Ghana Communication Technology University, Ghana, Ghana

The study examined how adolescents in Vanuatu use online platforms for health-related purposes. The survey of 197 students (urban and rural) revealed that 77% owned mobile phones, mainly for music and games. Only 24% used apps, social media, or websites for health, with Facebook, TikTok, YouTube, Instagram, and Twitter as main sources. Urban students preferred search engines, while rural students relied more on social media for health information. The findings challenge the digital divide narrative, emphasizing cultural sensitivity, misinformation risks, and the importance of policy and education in digital health engagement.

 The authors have made a valuable contribution by focusing on how youth utilize social media for healthcare information. However, I recommend strengthening the discussion section with supporting literature to substantiate the findings.

 In particular, it would be useful to further explain why the youth participants were not actively engaging with social media for healthcare information. Could this be attributed to their age, where critical health conditions are less prevalent, or perhaps to a general perception of invulnerability at this stage of life? Alternatively, could their priorities as students, such as education and social or entertainment-oriented activities limit their interest in health-related content? Clarifying these points would add depth to the interpretation of results.

 Additionally, it would be helpful to elaborate on the differences observed across the platforms or systems used for accessing health information, as this may provide further insights into patterns of engagement.

 I also encourage the authors to provide updated references. Specifically, the citation to Freeman et al. (2018) should be replaced with a current source. Similarly, in the statement beginning with *“This aligns with current literature that adolescents…”*, please support  your  claim with current literature. In this regard, you may consider replacing works such as Moreno et al. (2018) and Yonker et al. (2015) with current studies to strengthen the discussion.

If applicable, is the statistical analysis and its interpretation appropriate?

No

Is the study design appropriate and is the work technically sound?

Yes

Is the work clearly and accurately presented and does it engage with the current literature?

Partly

Are the conclusions drawn adequately supported by the results?

Partly

Are sufficient details of methods and analysis provided to allow replication by others?

Yes

Are all the source data and materials underlying the results available?

Yes

Reviewer Expertise:

My research focuses on social media use in healthcare, digital healthcare management systems, and telemedicine, with particular interest in how online platforms shape health communication, patient engagement, and access to care.

I confirm that I have read this submission and believe that I have an appropriate level of expertise to confirm that it is of an acceptable scientific standard, however I have significant reservations, as outlined above.

# Response

**Published:** 2025-09-12

**Authors:** Krestina Amon

**Affiliations:** Faculty of Medicine and Health, The University of Sydney, Sydney, New South Wales, Australia

**Discussion** We thank the reviewer for their positive feedback about the valuable addition of our findings in this space, and for their suggestions to improve our Discussion. To ensure that our arguments are based on our results, we have used our findings of the popular activities conducted by our participants as a potential reason for the limited engagement with social media for health information and supported with recent literature to provide greater depth to our interpretation of the results. We have also expanded our discussion to elaborate on the differences observed across the platforms and devices used for accessing health information. This includes discussing mobile phones, tablets and social media platform use supported by recent research literature. These additions help to clarify patterns of engagement and provide insight into how young people navigate digital health resources. **References** We have updated the manuscript accordingly to include more recent papers (including Armstrong et al., 2021; Black Dog Institute, 2024; Gazibara et al., 2024; Rich et al., 2020) and updated the text to align with the updated research papers cited.

# Reviewer report: Reviewer response for version 1

**DOI:** 10.21956/openreseurope.21277.r55609

**Published:** 2025-08-20

**Authors:** Rasika Manori Jayasinghe

**Affiliations:** 1 Prosthetic Dentistry, Penang International Dental College (PIDC), Butterworth, Penang, Malaysia; 2 Prosthetic Dentistry, University of Peradeniya, Peradeniya, Central Province, Sri Lanka

Results presentation is very poor. Total study is descriptive, does not add a big value to the literature. The results should have been analysed according to the independent variables such as age, gender, other socioeconomic factors...etc... 

 Discussion poorly written too more comparison and discussion on available literature should have been used for the discussion. you could get some guidance from : https://doi.org/10.1186/s12889-024-19008-5. Conclusions could have been improved if analysis was done. suggest major revision

If applicable, is the statistical analysis and its interpretation appropriate?

No

Is the study design appropriate and is the work technically sound?

Yes

Is the work clearly and accurately presented and does it engage with the current literature?

No

Are the conclusions drawn adequately supported by the results?

Partly

Are sufficient details of methods and analysis provided to allow replication by others?

Yes

Are all the source data and materials underlying the results available?

Yes

Reviewer Expertise:

OHRQOL, dental public health, prosthetic dentistry, implant dentistry

I confirm that I have read this submission and believe that I have an appropriate level of expertise to state that I do not consider it to be of an acceptable scientific standard, for reasons outlined above.

## References

[1] : Assessment of patterns and related factors in using social media platforms to access health and oral health information among Sri Lankan adults, with special emphasis on promoting oral health awareness. *BMC Public Health* .2024;24(1) : 10.1186/s12889-024-19008-5 10.1186/s12889-024-19008-5 38824505 PMC11143610

# Response

**Published:** 2025-09-12

**Authors:** Krestina Amon

**Affiliations:** Faculty of Medicine and Health, The University of Sydney, Sydney, New South Wales, Australia

**Results** We appreciate the reviewer’s suggestion to include further analysis of independent variables. While we agree that a stratified analysis can offer additional insights, our study was designed as an exploratory, descriptive investigation into adolescents’ use of online platforms for health information in Vanuatu. Given the limited number of participants who reported using digital tools for health purposes (n= 47; 24%), further disaggregation by age, gender, or socioeconomic status would not yield statistically meaningful results and could risk overinterpretation. We have clarified the scope and limitations of our descriptive approach in the Methods and Discussion sections, emphasising the value of this data in a context where little prior research exists. In addition, we have included subheadings to clearly present the findings as aligned with the research questions, as suggested by Reviewer 1 to provide a better structure to present the results. We believe, along with positive comments about our topic by Reviewers 1 and 3, that our study contributes meaningfully. It highlights digital health engagement patterns in an underrepresented population and identifies culturally specific factors that influence online health-seeking behaviour. **Discussion** We have reviewed the study suggested, which was published by the reviewer and colleagues on social media use for health and oral health information in Sri Lanka. It offers interesting insights into patterns of digital health engagement in a culturally and socioeconomically diverse context for those over 18 years of age. We developed our discussion based on the emerging themes from our results and incorporated relevant literature where available. However, we found that existing research on younger adolescents under 18 years in Pacific Island contexts is scarce, which limited our ability to draw on region-specific frameworks, but we drew on comparisons to the literature relating to the discussion topics where we could. We had added additional research citations based on suggestions from Reviewer 1. We believe our study contributes valuable insights and highlights the need for further research in this underrepresented region and age group and as such, have emphasised the importance of our study as a foundational contribution to this underexplored area in the Discussion. **Conclusion** We appreciate the reviewer’s recommendation to strengthen the conclusions through additional analysis. As noted earlier, our study was designed as a descriptive exploration of adolescents’ use of online platforms for health information in Vanuatu. Given the limited number of respondents who reported engaging with digital health tools (24%), we chose not to pursue inferential statistical analysis, as it would not yield reliable or generalisable results. Instead, we have revised the paper to better reflect the descriptive nature of the study while emphasising its contribution as foundational research in a context where digital health engagement among young adolescents in Vanuatu remains underexplored. We clearly articulate the implications for future research, policy development, and culturally sensitive health education strategies in Vanuatu and similar settings.

# Reviewer report: Reviewer response for version 1

**DOI:** 10.21956/openreseurope.21277.r54821

**Published:** 2025-07-02

**Authors:** Kay Oddone

**Affiliations:** 1 Charles Sturt University, Albury, New South Wales, Australia

**Title and Abstract**

 The title has some alignment with the scope of the research, however the focus on social media is somewhat misleading, as the paper actually explores many different aspects of adolescents’ engagement with digital information, with particular attention to digital health information. The abstract presents the aim, methods, key results, and conclusions clearly and concisely. It reflects the paper’s content and signals its relevance to adolescent digital health literacy.

**Introduction and Literature Review**

 The introduction sets the context but contains several unsupported claims. For example, statements on adolescents’ media use, links between usage patterns and educational initiatives, and disparities in urban digital access require citations. Core terms such as *digital health landscape*, *health information-seeking behaviours*, and *digital health literacy* appear without definition. Defining these terms and grounding assertions in current scholarship will strengthen the argument and establish conceptual clarity. To do so, I recommend consulting paper such as

1. Merga, M. K. (2024). - **Ref 1. **
2. Taba, M., et.al. (2022). **Ref 2**
3. Oddone, K., & Merga, M. (2025)- **Ref 3**.

In addition, the introduction would be improved by clear presentation of the research questions as a series of dot points rather than embedded within the text. This would help the reader make stronger and more immediate connections with the title of the paper as well as with the sections of the findings, as the wording of the questions could be more easily identified within the findings being reported.

**Methodology**

 The research design is appropriate, yet important details are missing. Specify student age ranges in addition to grade levels to aid international readers. It would be useful to describe any teacher training provided to minimise survey administration bias. This is particularly important given the context of the research, where students may be relying on teachers to aid their understanding, and teachers may not be familiar with the importance of unbiased guidance. Also please consider including the full survey as an appendix to enable replication. These additions will enhance rigour and transparency.

**Findings and Discussion**

 The presentation of findings would benefit from greater clarity and precision, particularly in the use of the term's *social media* and *platform*. For instance, when discussing the responses to the survey question, “Which social media platform/site is your favourite?” identifying that a high number of responses were “social media,” is not helpful as the phrasing of the question likely prompted respondents to remain within the bounds of the term provided. The more relevant reporting was concerning the responses identifying specific platforms, which offer more meaningful insights into participants’ actual engagement behaviours. Refinement of the representation of the findings in this way would improve the impact of the study for readers. Consider using subheadings rather than bolded text through the findings to aid comprehension and relation of the findings to the original research questions. Also consider listing the results using dot points to aid perusal of the findings.

 The discussion integrates results with prior work and offers practical advice to improve adolescents’ digital health literacy. It would benefit from explicit links back to each research question and deeper reflection on how risks including misinformation and passive exposure could be mitigated. It is also not evident why Cultural sensitivity is not considered a ‘risk’ and grouped with the other concerns.  

 The limitations and future research directions mainly discuss limitations, without offering directions for future research and possible strategies to address the low response rate. Consider elaborating on this section to demonstrate where and how this research will be expanded, as the findings indicate there is more work to be done in this area.

**Conclusion**

 The conclusion summarises the study but could tie findings more explicitly to the defined concepts and broader educational implications. It is also concerning that a major conclusion is that there is a preference for social media use, when some questions only offered social media platforms as options to choose for their favourite information source. Perhaps the conclusion should be narrowed to the specific platforms students used, or the preference for mobile phone over other devices. A brief restatement of contributions and recommended next steps would improve coherence.

**Overall Strengths**

- Clear, concise abstract
- Well-motivated research topic
- Practical focus on improving adolescent digital health literacy

**Areas for Improvement**

- Provide references for all key claims in the introduction.
- Define *digital health landscape*, *health information-seeking behaviours*, and *digital health literacy* early.
- Add age ranges, teacher training details, and the survey instrument to the methods section.
- Strengthen the discussion by connecting findings to research questions and suggesting future studies.

**Recommendation**

*Minor revisions* – The study addresses an important issue and uses a suitable design, but it needs additional references, clearer definitions, and fuller methodological detail before indexing.

If applicable, is the statistical analysis and its interpretation appropriate?

Yes

Is the study design appropriate and is the work technically sound?

Yes

Is the work clearly and accurately presented and does it engage with the current literature?

Partly

Are the conclusions drawn adequately supported by the results?

Partly

Are sufficient details of methods and analysis provided to allow replication by others?

Partly

Are all the source data and materials underlying the results available?

No

Reviewer Expertise:

Education, digital literacy, information literacy.

I confirm that I have read this submission and believe that I have an appropriate level of expertise to confirm that it is of an acceptable scientific standard, however I have significant reservations, as outlined above.

## References

[1] : TikTok and digital health literacy: A systematic review. *IFLA Journal* .2025;51(2) : 10.1177/03400352241286175 490-501 10.1177/03400352241286175

[2] : Adolescents’ self-efficacy and digital health literacy: a cross-sectional mixed methods study. *BMC Public Health* .2022;22(1) : 10.1186/s12889-022-13599-7 10.1186/s12889-022-13599-7 35725389 PMC9207829

[3] : Evaluation Strategies of School Students Accessing Health Information in Social Media Videos: A Case Study Investigation. *Journal of Library Administration* .2025;65(2) : 10.1080/01930826.2024.2437964 214-234 10.1080/01930826.2024.2437964

# Response

**Published:** 2025-09-12

**Authors:** Krestina Amon

**Affiliations:** Faculty of Medicine and Health, The University of Sydney, Sydney, New South Wales, Australia

**Title and abstract** We thank the reviewer for their comments about the title. The title has been amended to state the use of online platforms, rather than single out social media specifically. **Introduction and Literature Review** We have had a closer look at the Introduction and believe that the claims in the first paragraph about adolescents' use of social media are all currently supported with citations from Hausmann et al., 2017; Moreno et al., 2018; Gray et al., 2005; Skinner et al., 2003.

 For clarity, the existing Caillaud et al., (2022) citation is the evidence to support the claim of "adolescents engage and respond well to tailored digital education programs targeting health-related behaviour such as physical activity in Australia…" and the Galy et al., (2019) citation for the research on this in the Pacific Islands. Furthermore, the existing Nedjar-Guerre et al., (2023) citation is the research evidence to support the claim of disparities in urban digital access.

 We appreciate the reviewer's suggestion regarding defining the term "digital health landscape." This term has been widely used in existing literature without a standardised definition, often serving as a broad conceptual umbrella encompassing the use of digital technologies and systems influencing information delivery, access, and innovation. However, to improve clarity for readers less familiar with this terminology, we have now included further description within our sentence.

 While we recognise the importance of conceptual clarity relating to defining "information-seeking behaviours", we’ve opted not to include a formal definition of information-seeking behaviours as we believe the term is self-explanatory, and often used, as well as well-established in the literature and generally understood within the context of engagement. The discussion throughout the manuscript implicitly defines and illustrates the concept through examples of online searches, social media interactions, and peer-to-peer information exchange. We have however, amended the sentence so that the references cited for this is made more explicit that these papers discuss adolescent information-seeking behaviours.

 In the original manuscript, a definition of digital health literacy "represents the capacity to proficiently navigate health-related information and resources within digital environments" is already provided and is supported with a citation from Schulenkorf et al. (2021). We have reviewed the definition in the reviewer's published paper as suggested, and believe that the definition we have provided is aligned. We have updated this to include a citation of the reviewer’s work for our definition.

 The Taba et al., (2022) paper that the reviewer suggested was already cited in the same paragraph relating to digital health literacy.

 The paper by the reviewer's colleague, Merga (2025), was useful and has been included in the Discussion section of the paper, relating to risks.

 The new suggested citations of the reviewer's work and colleague have been included in the reference list of the updated version of the paper.

 As suggested by the reviewer, we have updated the presentation of our research questions in point form in version 2 of the paper. **Methodology** In the updated version of the paper, we have included i) the age range of the participants (ie. 14-20 years of age) in the Results section, and ii) a description of instructions to the teachers (ie. "To minimise bias in the administration of the questionnaire, the teachers were instructed that they were allowed to provide definitions of any words within the questionnaire that the participant may not have understood, but were asked not to provide any examples that may suggest answers to the questions"), in the Methods section.

 As required by the Journal, the questionnaire questions have already been provided in the data availability section and cited (Galy et al., 2024a; Galy et al., 2024b). **Findings and Discussion** We understand the concern raised by the reviewer and have modified the description of the Results in version 2 of the paper to better present the findings. We have also updated each of the Figures to better present the social media platforms identified by the participants. And included a Supplementary Table to clearly present the analysis of responses. To further provide links with the research questions, we have included subheadings to more clearly present the findings as aligned with the research questions.

 Within the Discussion section, to provide more details on the risks we have identified, we have included the work of your colleague, Merga (2024) and the work of the reviewer (Oddone & Merga, 2025) to provide more details on how the risk of misinformation and exposure could be mitigated. Regarding the reviewer's observation regarding cultural factors and their relation to risk in adolescent social media use for health purposes, in our paper we conceptualise cultural sensitivity as a contextual and structural consideration, rather than as a discrete risk factor. While cultural dynamics can amplify or mitigate digital health risks—such as exposure to misinformation or stigma—we have framed culture as a cross-cutting influence that informs adolescent engagement and the effectiveness of health communication strategies. Therefore, rather than positioning culture as a risk in itself, our approach emphasises its role in shaping digital health literacy, access, and relevance. We hope this framing provides a more holistic understanding and avoids reinforcing deficit-based interpretations of cultural identity. After review of the Limitations and Future Research Directions section, we agree that we lacked suggestions for future research. We have updated this with suggestions on moving forward. **Conclusion** Based on reviewer feedback we have amended the Conclusion section to include what our study contributes to, providing more specific identification of social media platforms from our results, and a final statement of further research on this topic.
